# A randomized clinical study to evaluate the possible antifibrotic effect of zinc sulfate in chronic HCV patient receiving direct-acting anti-viral therapy

**DOI:** 10.1007/s10787-024-01628-3

**Published:** 2025-01-09

**Authors:** Sahar M. El-Haggar, Dina S. Attalla, Mostafa Elhelbawy, Dalia R. El-Afify

**Affiliations:** 1 Department of Clinical Pharmacy, Faculty of Pharmacy, Tanta University, El-Gharbia Government, Tanta, Egypt; 2 Faculty of Pharmacy, Menoufia University, Shebin El-kom, Egypt; 3 Clinical Pharmacy Unit, National Liver Institute, Menoufia University, Shebin El-Kom, Egypt; 4 Department of Hepatology and Gastroenterology, National Liver Institute, Menoufia University, Shebin El-Kom, Egypt

**Keywords:** Zinc, Hyaluronic acid, Fibronectin, Liver fibrosis, Transforming growth factor beta 1

## Abstract

**Objective:**

This study aimed to assess the potential antifibrotic impact of zinc sulfate in chronic Hepatitis C Virus (HCV) patients receiving direct-acting antiviral therapy.

**Methods:**

This randomized controlled study included 50 chronic HCV-infected patients with fibrosis stage (F1 & F2). Participants were randomly assigned to two groups: Group 1 (Control group, *n* = 25) received standard direct-acting antiviral therapy for 3 months, while Group 2 (Zinc group, *n* = 25) received 50 mg/day of zinc sulfate in addition to the standard direct-acting antiviral therapy for the same duration. Baseline and 3-month post-intervention assessments included evaluating serum levels of hyaluronic acid, transforming growth factor beta-1, and fibronectin. Furthermore, indices of liver fibrosis, such as the Fibrosis Index based on the 4 factors (FIB-4) and the Aspartate Transaminase-to-Platelet-Ratio Index (APRI), were calculated during these assessments.

**Results:**

At baseline, the two studied groups had no statistical difference in demographic and laboratory data. After treatment, serum zinc levels significantly increased in the zinc-treated group compared to the control group. Additionally, serum fibronectin and hyaluronic acid levels were significantly reduced in group 2 (zinc group) compared to group 1 (control group). Moreover, zinc group showed lower APRI scores than the control group after a 3-month follow-up period, but there was non-significant difference in FIB-4 scores between the two groups after treatment. Furthermore, total bilirubin levels were reduced after zinc therapy for 3 months.

**Conclusions:**

Administering zinc sulfate could potentially serve as a safe and efficient therapeutic strategy for the management of hepatic fibrosis in individuals with chronic hepatitis C virus.

**Trial Registration:**

ClinicalTrials.gov identifier: NCT05465434, On 19/7/2022.

## Introduction

Infection with the hepatitis C virus has become a major worldwide health concern. According to the World Health Organization's 2017 report, approximately 71 million people worldwide are afflicted with chronic HCV infection. Notably, Egypt bears the highest prevalence of HCV infection, ranging from 6 to 40% (Naga et al. 2019).

Viral hepatitis is estimated to rank as the seventh leading cause of mortality on a global scale. Hepatitis C virus is directly responsible for almost half of this mortality and the main cause of liver fibrosis, cirrhosis, and cancer development (Kouyoumjian et al. 2018).

Liver fibrosis is a serious sequela of chronic insult to the liver from any etiology. The replacement of tissue by a collagenous scar brought on by recurrent liver injuries is known as fibrosis and it has a spectrum that extends from non-cirrhotic (stages F0–F3) to cirrhotic (stage F4) (Khatun et al. 2019).

With the development of direct-acting antiviral medications, or DAAs, the management of chronic hepatitis C virus infection experienced a revolution. Combining sofosbuvir 400 mg and daclatasvir 60 mg has been linked to a high percentage of SVR (sustained virological response) in patients with genotypes 1 or 4, who are thought to be difficult to treat (Ahmed et al. 2018).

Serum zinc levels in healthy controls were found to be higher than those in people with asymptomatic HCV carriers and chronic liver disease associated to HCV (Himoto et al. 2018).

Zinc (Zn) is a trace element and cofactor for several enzymes and proteins that is crucial to biological processes involving cell division, growth, and development as well as cellular integrity (Himoto et al. 2018).

This study aimed to assess the potential antifibrotic impact of zinc sulfate in chronic HCV patients undergoing direct-acting antiviral therapy. The evaluation involved measuring serum levels of key biomarkers associated with liver fibrosis, including fibronectin (FN), hyaluronic acid (HA), and transforming growth factor beta-1 (TGF-β1). Additionally, indices quantifying liver fibrosis, such as the Fibrosis Index based on the 4 factors (FIB-4) and the Aspartate Transaminase-to-Platelet Ratio Index (APRI), were calculated as part of the comprehensive assessment (Mostafa et al. 2021).

## Patients and methods

### Study design and patient selection

Fifty individuals with fibrosis stage F1(mild fibrosis), F2 (moderate fibrosis) and chronic hepatitis secondary to HCV infection were involved in this prospective randomized controlled research. The patients were enrolled between August 2022 and September 2023 from the Hepatitis C Unit at the National Liver Institute of Institutional University Hospital in Shebin El-Kom, Menoufia Governorate, Egypt.

The study adhered to ethical standards outlined in the 1964 Declaration of Helsinki and its subsequent amendments, obtaining approval from Tanta University's and Menoufia University's Institutional Research Ethics Committees. All subjects gave written informed consent prior to their inclusion in the study, and the study was registered as a clinical trial (ClinicalTrials.gov identifier: NCT05465434).

In accordance with the Consolidated Standards of Reporting Trials (CONSORT) criteria, participants were randomized at random in a 1:1 ratio using computer-generated codes. Two groups were assigned to them: Group 1 (control group, *n* = 25) underwent a 3-month course of conventional medication consisting of 400 mg/d of sofosbuvir combined with 60 mg/d of daclatasvir. Also, for 3 months, Group 2 (the Zinc group, *n* = 25) got both conventional DAAs therapy and an oral dose of 50 mg zinc sulfate per day.

Fibrosis diagnosis was based on clinical and laboratory data, and patients were categorized as F1 or F2 according to FIB-4 and APRI scores.

Inclusion criteria encompassed individuals aged 18 to 65 years with chronic HCV infection and liver fibrosis stages F1 and F2. On the other hand, hepatitis B virus infection (HBV), HIV, history of hepatocellular carcinoma (HCC), previous liver transplantation, hypotension, cardiomyopathy, pregnancy, lactation, thalassemia, acute hepatitis, renal dysfunction (creatinine > 1.5 mg/dl, creatinine clearance < 40 ml/min), portal vein thrombosis, and diabetes were among the exclusion criteria.

Comprehensive evaluations were carried out for both study groups at baseline and three months following the start of medication, including complete blood count, liver function tests, FIB-4 score, APRI computation, and biomarkers of liver fibrosis.

### Measurement of liver function parameters

Serum alanine aminotransferase (ALT) and serum aspartate aminotransferase (AST) were determined spectrophotometrically using the kinetic method (Chinnappan et al. 2023). The colorimetric (Diazo) approach was utilized to perform a spectrophotometric test of the levels of total and direct serum bilirubin (Narwal et al. 2021). Spectrophotometric analysis was used to quantify the content of serum albumin using a modified bromocresol green colorimetric approach (Xu et al. 2022a, 2022b). (Siemens Healthcare Diagnostics Products Gmbh, Marburg, Germany), provided the kits utilized for the biochemical analysis.

### Analysis for biomarkers of liver fibrosis

Serum HA, TGF-β1, and fibronectin levels were measured by double antibody sandwich ELISA, which was performed in compliance with the manufacturer's instructions (Sun Red; Sun Red Biomedical Technology Co Ltd, Shanghai, China) and utilized commercially available ELISA kits.

### Analysis of serum zinc level

Serum zinc level was measured using a colorimetric 5-Bromo-PAPS test following the manufacturer's instructions and using commercially available zinc assay kits (Egyptian Company for Biotechnology S.A.E, Cairo, Egypt).

### Computation of liver fibrosis indices

The following formulas were used to calculate the liver fibrosis indices, namely the fibrosis index based on the 4 factors (FIB-4) and the Aspartate Transaminase-to-Platelet Ratio Index (APRI):

- Age (years) × AST (IU/L)/ (platelet count (× 10^9^ /l) × √ALT (IU/l)) is the formula for the FIB-4 score. The FIB-4 index uses cut-off values of 3.25 and 1.45, respectively, to rule in or out severe fibrosis. In 82% of patients, a FIB-4 score more than 3.25 indicates the presence of advanced fibrosis (F4), whereas in 94.7% of patients, a FIB-4 score less than 1.45 indicates the absence of advanced fibrosis (F3-F4) (Xu et al. 2022a, 2022b).
- (AST (IU/L)/ upper limit of normal) × 100/platelet count (10^9^/L) is the formula of APRI. The APRI index uses 0.5 as the cut-off value. A score of less than or equal to 0.5 indicates that the liver has either minimal scarring (F1 or F2 according to French METAVIR Score) or is completely devoid of fibrosis (F0) (Cheng et al. 2020).

### Statistical evaluation

The statistical software IBM-SPSS version 28.0 was used to analyze all the data, which were displayed as mean ± SD. Shapiro–Wilk test was used to check the normality of each data variable. To assess the differences in categorical data between the two groups, the chi-square test was applied. Mann–Whitney U test or an independent samples t test was used to compare continuous data between the two groups. To compare continuous data within the same group before and after administering the drug, a paired t test or Wilcoxon signed rank was employed. Spearman correlation test was used to perform correlation analysis. This study's statistical tests were all two-tailed and performed with a predefined significance threshold of 0.05.

### Sample size calculations

Group sample sizes of 14 and 14 achieve 80% power to detect superiority in reducing fibronectin level using a one-sided, two-sample t test. The margin of superiority is 10.0. The true difference between the means is assumed to be 50.0. The significance level (alpha) of the test is 0.050. The data are drawn from populations with standard deviations of 41.3 and 41.3.

Hence, sample size is 18 accounting for 20% attrition rate or may be more (Ghafar et al. 2019).

## Results

**Fig. 1:**
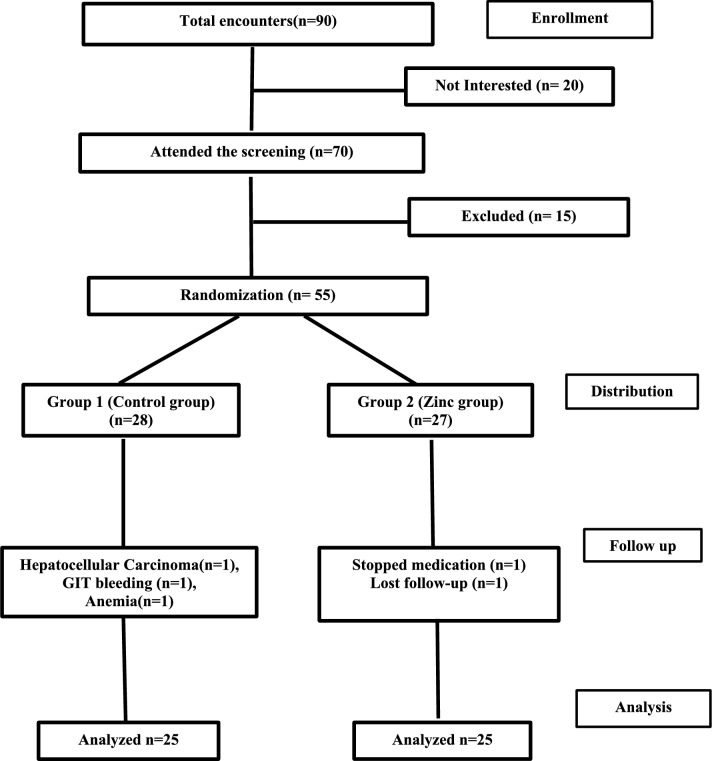
Flowchart of participants

Ninety patients with chronic HCV liver fibrosis were screened; twenty of them declined to participate, and fifteen were ruled out of the study due to exclusion criteria. The remaining 55 patients were enrolled and randomized to one of the two study groups based on their fulfillment of the inclusion criteria. Five patients were dropped from the study throughout the follow-up period. Hence, they were not included in the final analysis. The reasons for dropout included anemia (1 patient), loss of follow-up (1 patient), medication cessation (1 patient), development of hepatocellular carcinoma (1 patient), and severe gastrointestinal bleeding (1 patient). Consequently, the trial was completed successfully by just 50 patients whose data were included in the final analysis. Figure 1 shows the flowchart of participants.

In Table 1, the demographic and CBC data of group 1 (control); who received the conventional DAAs therapy of HCV, which is sofosbuvir 400 mg and daclatasvir 60 mg, and group 2 (zinc); who received the conventional DAAs therapy and 50 mg of zinc sulfate, showed non-significant differences. Also, the baseline liver and kidney functions in Table 2 and baseline liver fibrosis markers and zinc level in Table 3 indicate that there was a non-statistically significant difference (*P* > 0.05) in all study participants between the two research groups.

**Table 1:** Change in demographic data and CBC within and between the two study groups

Parameter	Group 1: Control (*n* = 25)			Group 2: Zinc (*n* = 25)			
	Before treatment	3 months after treatment	^*a*^*p*-value	Before treatment	3 months after treatment	^*a*^*p*-value	^*b*^*p-*value after treatment
BMI	25.49 ± 1.85	26.22 ± 1.66	0.137	26.37 ± 2.47	26.79 ± 2.49	0.236	0.35
WBCs (*10^3^/mm^3^)	8.4 ± 2.8	8.4 ± 2.3	0.893	7.7 ± 2.6	8.2 ± 2.2	0.412	0.69
Hb (gm/dl)	13.3 ± 1.9	13.0 ± 1.6	0.272	14.1 ± 1.7	12.8 ± 1.6	0.006*	0.722
Platelets (*10^3^/mm^3^)	275.2 ± 55.8	265.3 ± 55.9	0.216	267.9 ± 60.1	268.4 ± 37.2	0.84	0.567
INR	1.05 ± 0.08	1.03 ± 0.07	0.38	1.09 ± 0.12	1.08 ± 0.06	0.64	0.026*
PT sec	12.56 ± 0.51	12.16 ± 0.75	0.02*	12.36 ± 0.49	12.52 ± 0.59	0.29	0.064

*BMI* body mass index; *WBCs*  white blood cells; *Hb* hemoglobin; *INR*  international normalized ratio; *PT*  prothrombin time. The changes are expressed as mean ± SD. a, b: level of significance within and between groups respectively; * Indicates a significant *p*-value at the 0.05 level, In the within-group comparisons

**Table 2:** Change in Liver and Kidney Function within and between the two study groups

Parameter	Group 1: Control (*n* = 25)			Group 2: Zinc (*n* = 25)			
	Before treatment	3 months after treatment	^*a*^*p*-value	Before treatment	3 months after treatment	^*a*^*p*-value	^*b*^*p*-value after treatment
ALT (IU/L)	42.1 ± 20.3	20.8 ± 10.6	< 0.001*	45.0 ± 35.2	25.1 ±	< 0.001*	0.197
AST (I/UL)	34.5 ± 10.9	21.4 ± 8.1	< 0.001*	29.8 ± 14.7	22.9 ± 9.7	0.011*	0.662
BIL-T (mg/dl)	0.67 ± 0.4	0.70 ± 0.5	0.686	0.54 ± 0.2	0.46 ± 0.3	0.042*	0.041*
BIL-D (mg/dl)	0.26 ± 0.28	0.57 ± 0.27	0.002*	0.21 ± 0.17	0.50 ± 0.23	< 0.001*	0.362
Albumin(g/dl)	4.41 ± 0.38	4.47 ± 0.37	0.62	4.55 ± 0.32	4.39 ± 0.46	0.15	0.509
BUN (mg/dl)	14.28 ± 4.06	11.68 ± 4.04	0.01*	14.40 ± 4.84	11.40 ± 2.92	0.02*	0.78
SCr (mg/dl)	0.8 ± 0.2	0.8 ± 0.1	0.944	0.7 ± 0.2	0.8 ± 0.2	0.306	0.418

*ALT* alanine aminotransferase; *AST* aspartate transaminase; *BIL-T* total bilirubin; *BIL-D* direct bilirubin; *BUN* blood urea nitrogen; *SCr* serum creatinine

The changes are expressed as mean ± SD

a, b: level of significance within and between groups respectively

^*^Indicates a significant *p*-value at the 0.05 level, In the within-group comparisons

**Table 3:** Change in Zinc level and Liver fibrosis biomarkers within and between the two study groups

Parameter	Group 1: Control (*n* = 25)			Group 2: Zinc (*n* = 25)			
	Before treatment	3 months after treatment	^*a*^*p*-value	Before treatment	3 months after treatment	^*a*^*p*-value	^*b*^*p*-value after treatment
Zinc(µg/dl)	65.8 ± 13.8	67.9 ± 14.5	0.628	62.4 ± 16.8	101.9 ± 12.2	< 0.001*	< 0.001*
Fibronectin(ng/ml)	151.1 ± 16.7	206.8 ± 74.8	0.001*	157.9 ± 27.2	167.6 ± 34.5	0.23	0.023*
HA (ng/ml)	53.8 ± 14.0	59.8 ± 14.2	0.091	53.9 ± 9.6	51.7 ± 10.6	0.434	0.026*
TGF- β_1_ (ng/ml)	9.6 ± 3.6	11.4 ± 4.7	0.206	8.4 ± 4.7	9.9 ± 2.6	0.201	0.183
FIB4	0.72 ± 0.41	0.72 ± 0.31	0.721	0.63 ± 0.40	0.63 ± 0.30	0.563	0.187
APRI	0.40 ± 0.21	0.29 ± 0.09	0.192	0.33 ± 0.23	0.20 ± 0.11	0.048*	0.023*

*HA* hyaluronic acid; *TGF-β*_*1*_ transforming growth factor-b1; *FIB-4*  fibrosis index based on the 4 factors; *APRI* aspartate transaminase-to-platelet ratio index

The values are shown as *n* (%)

a, b: level of significance within and between groups respectively; * Indicates a significant *p*-value at the 0.05 level, In the within-group comparisons

*P* < 0.05 is considered significant

But AST and ALT levels were significantly decreased in control group and zinc group after treatment, bilirubin level significantly decreased within zinc group and between the two study groups after treatment as shown in Table 2.

As stated in Table 3, within groups, serum zinc levels were significantly increased after 3 months in zinc group patients compared to its baseline value (before treatment), However, the APRI score significantly dropped in the zinc group compared to the baseline values. Nevertheless, no significant changes were observed in fibronectin, hyaluronic acid, TGF-β1 and FIB-4 after 3 months of zinc treatment compared to its baseline value. Fibronectin levels significantly increased in the control patients after 3 months compared to the baseline levels. However, no significant changes were observed in hyaluronic acid, TGF-β1, FIB-4, and APRI score after 3 months compared to its baseline value in the control group.

On the other hand, after three months of administering the medication, the zinc group's serum zinc level was significantly higher than the control's. Additionally, serum fibronectin and hyaluronic acid levels were significantly reduced with zinc group compared to the control. Moreover, zinc group patients showed lower APRI scores than the control group after a 3-month follow-up period. Furthermore, zinc group had a lower but non-significant difference in the level of TGF-β1 and FIB-4 score compared to the control group after 3 months of zinc treatment.

**Fig. 2:**
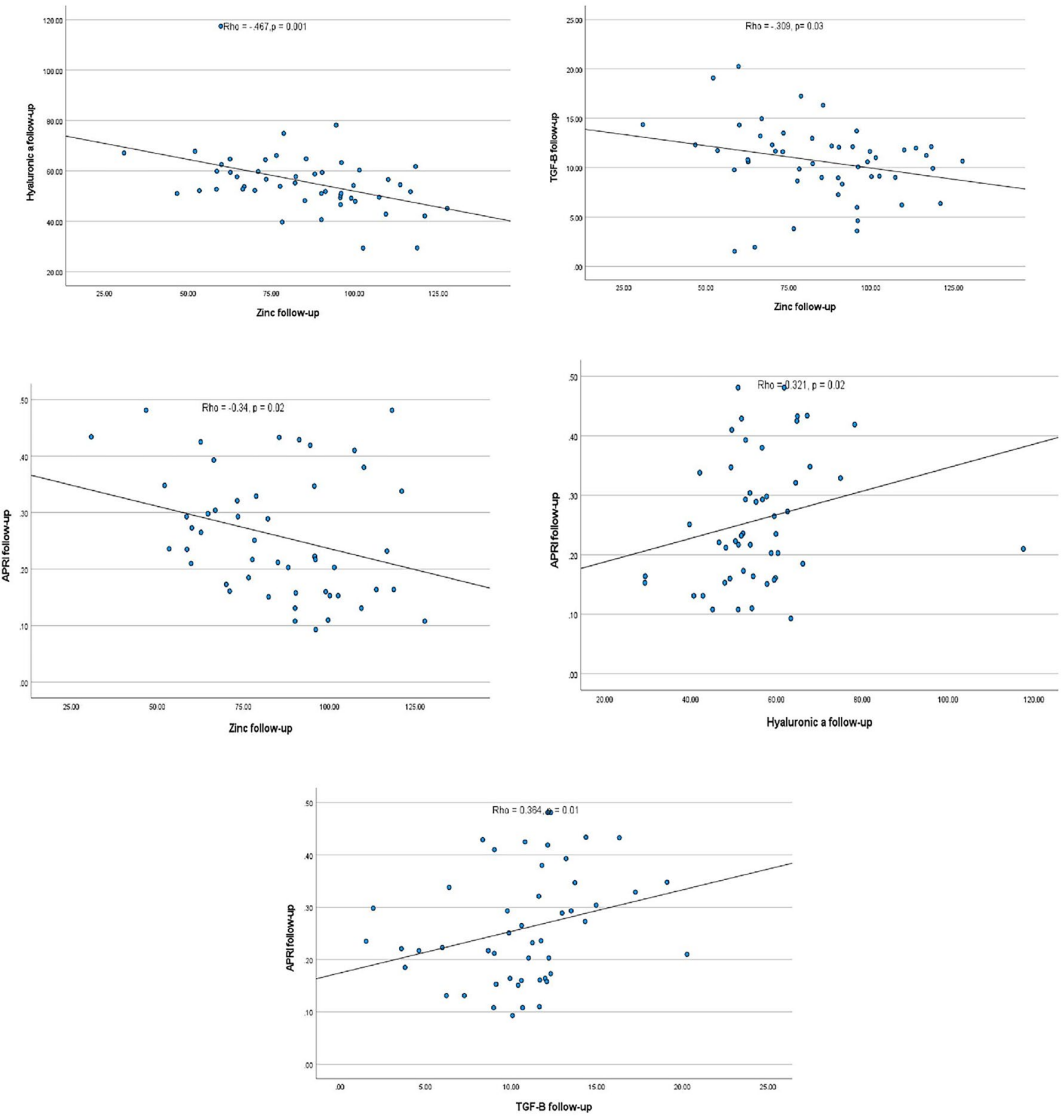
Spearman correlation analysis between the measured variables TGF-β1 for transforming growth factor-b1; APRI for aspartate transaminase-to-platelet ratio index

Significant negative correlations were found between zinc level and each of hyaluronic acid and TGF- β1 levels. Additionally, the APRI score had a significant negative correlation with zinc level and significant positive correlations with each of hyaluronic acid and TGF-β1, as presented in Fig. 2.

### The study drugs' tolerability and safety

No participants in the study groups experienced any significant adverse responses linked to medication-associated problems. Compared to two patients in the zinc group (*P* = 1.00), just one patient in the control group had arrhythmia.

Throughout the study period, nobody in the control group experienced hypoglycemia, cough, or anemia (a drop in hemoglobin level of at least 2 g/L) during the study period. Controllable nausea and vomiting occurred in 13 patients in the control group compared to 18 patients in the zinc group (*P* = 0.24), mild abdominal pain occurred in 10 patients in the control group compared to 13 patients in the zinc group (*P* = 0.57), and serum creatinine elevated in 5 patients in the control group compared to 3 patients in the zinc group (*P* = 0.7).

## Discussion

In 2002, the World Health Organization declared that zinc deficiency stands as one of the foremost risk factors contributing to morbidity and mortality, particularly in developing countries **(**Ozeki et al. 2020**)**. Poor survival in chronic viral patients with early HCC was significantly associated with Zn deficiency (Kim et al. 2020).

Chronic liver disease (CLD) is among the conditions associated with diseases that can lead to zinc deficiency in adults (Ozeki et al. 2020).

Zinc deficiency in chronic liver diseases arises from a combination of factors, including a reduced capacity to synthesize albumin, malabsorption of zinc from the intestine, and an elevated excretion of zinc in the urine (Himoto et al. 2020; Ozeki et al. 2020; Grüngreiff et al. 2016; Fathi et al. 2020).

Previously reported RCTs demonstrated the potential of zinc supplementation to ameliorate hepatic encephalopathy-related clinical symptoms, as well as to enhance clinical parameters (such as blood albumin, ammonia levels, and Child–Pugh score) or to stop the evolution of the disease (e.g., progression to HCC) (Diglio et al. 2020; Shipley et al. 2019).

Previous clinical studies have shown that polaprezinc (a protector of the gastric mucosa) has been recently demonstrated to be an inhibitor of liver fibrosis in a mouse model suggesting that it may have antifibrotic effect in people suffering from autoimmune hepatitis and liver cirrhosis as it may affect collagen degradation through deactivation of hepatic stellate cells (HSCs) by inhibiting both cell behavior transitions and essential gene production and also modulation of the activity of matrix metalloproteinases (MMPs) and their tissue inhibitors of metalloproteinase (TIMs) (Ye et al. 2017; Moriya et al. 2021; Takahashi et al. 2007).

We aimed in the present study to focus on the potential antifibrotic impact of zinc sulfate in chronic HCV patients with mild and moderate fibrosis receiving direct-acting anti-viral therapy.

Zinc supplements influence hepatic fibrosis by decreasing the elevated fibrogenic activity in late stages of liver injury and increasing hepatic collagenolysis which is suppressed in early stages of liver fibrosis (Giménez et al. 1994).

Zinc supplementation results in a significant reduction in collagen deposition in the liver. Furthermore, zinc suppresses gene expression of α-SMA and collagen I and enhances the capacity of collagen degradation as determined by the increased activity of total collagenases and elevated mRNA and protein levels of MMP13, so zinc supplementation suppresses liver fibrosis through both inhibiting collagen production and enhancing collagen degradation (Shi et al. 2015).

Zinc supplementation attenuated the increase in factors known to be associated with hepatic apoptosis (Zhou et al. 2008; Kang et al. 2008).

Zinc deficiency causes a decline in the activity of collagenase that leads to liver fibrosis (Stamoulis et al. 2007; Seltzer et al. 1977). Moreover, zinc exerts membrane-stabilizing activity on liver lysosomes and has cytoprotective activities that protect hepatocytes from oxidative stress (Zhou et al. 2005; Stamoulis et al. 2007; Kono et al. 2012).

In the present study we used the standard dose of zinc sulfate that provides the patients with 50 mg of elemental zinc, which represent the daily requirement of the human body for 3 months which matches some previous clinical studies (Somi et al. 2012; Mohammad et al. 2012; Shirahashi et al. 2021; Hosui et al. 2021).

Despite being one of the most accurate ways to evaluate the condition of the liver, liver biopsies are an intrusive procedure and may lead to severe complications, bleeding and hemodynamic instability (Chowdhury et al. 2023; Boyd et al. 2020).

Consequently, we employed safe and noninvasive techniques to measure liver fibrosis in this study, such as measuring serum levels of liver fibrosis biomarkers (i.e., HA, FN and TGF-β_1_) and calculating FIB-4 and APRI scores. It has been shown that HA, FN and TGF-β_1_ are the most useful diagnostic and prognostic noninvasive biomarkers for hepatic fibrosis (Ghafar et al. 2019; Boyd et al. 2020; Ehsan et al. 2021; Rewisha et al. 2021; Dewidar et al. 2019). Additionally, it was observed that the FIB-4 and APRI scores had a diagnostic precision for determining the liver fibrosis stage (Itakura et al. 2021).

In the current study, the administration of zinc sulfate showed a significant improvement in serum zinc level and a remarkable decline in the liver fibrosis index (APRI score) and biomarkers (HA and FN) when compared to the control group. Bilirubin level significantly decreased within zinc group and between the two study groups after treatment as revealed in previous studies which indicate the positive effect of zinc on the liver (Hiraoka et al. 2020).

The phases of liver fibrosis range from non-cirrhotic stages (F0–F3) to cirrhotic stage (F4). Fibrosis, characterized by the replacement of parenchyma with a collagenous scar due to repetitive liver hits, is influenced by different etiologic agents including diabetes, alcohol intake, metabolic syndrome, viral infections like HCV and many other diseases (Shirahashi et al. 2021; Masuzaki et al. 2020; Grüngreiff et al. 2016).

Opposing the traditional view that it is a unidirectional process, several research studies have demonstrated liver fibrosis's dynamic nature and potential for reversibility. Preventing, decreasing and reversing the progression of fibrosis to cirrhosis and its related problems is the main goal of current and future therapy for chronic liver disease, this will lessen the need for liver transplantation (Himoto et al. 2020; Iritani et al. 2022).

The liver plays a crucial role in maintaining the body's zinc homeostasis. Numerous chronic liver diseases, such as nonalcoholic fatty liver disease, chronic viral hepatitis, and liver cirrhosis, have been linked to zinc deficiency. Such deficiency can lead to the impairment of multiple hepatic functions. Furthermore, studies have indicated a correlation between zinc deficiency and liver fibrosis in chronic HCV patients (Omran et al. 2017).

Liver fibrosis occurs due overproduction of extracellular matrix (ECM) which consists of substances including glycoproteins, collagen, proteoglycans, and glycosaminoglycans.

Increased production and accumulation of extracellular matrix in liver tissues affect the normal liver cell functions and increases the progression of fibrosis. The concentrations of ECM in blood serve as biomarkers of fibrotic diseases (Matsuda et al. 2020).

HCV replication and proliferation activate immune cells in the liver which are for example, Kupffer cells, macrophages and natural killer cells that leads to activation of hepatic stellate cells (HSC) that secrete fibrillar collagens (Shipley et al. 2019). In hepatic fibrosis, liver sinusoidal endothelial cells are also beneficial and essential cells. These cells have the capacity to activate HSC and produce fibronectin in the very early stage of liver damage.

Fibronectin is non-collagenous glycoprotein fundamental constituent of ECM and is considered a reliable marker of liver fibrosis (Acharya et al. 2021).

In our study, fibronectin level was significantly higher in the control group; however, there was no significant difference in fibronectin level in the zinc group. In addition, administration of zinc sulfate caused a significant decrease in serum fibronectin levels in zinc group compared to the control which supports a prior study that found zinc reduces the gene expression of liver fibrotic markers, such as fibronectin in vitro. (Ye et al. 2017; Shan et al. 2023).

The most important and fundamental glycosaminoglycan of extracellular matrix (ECM) is hyaluronic acid (HA), which is primarily produced by hepatic stellate cells and broken down by sinusoidal endothelial cells. According to previous studies, it may correlate with the histological phases of liver fibrosis in chronic liver disorders (Takahashi et al. 2007).

In our clinical research, zinc sulfate caused a significant decrease in serum hyaluronic acid levels in zinc group compared to the control and compared to their baseline value, which agrees with a previous study that zinc level is negatively correlated with hyaluronic acid level in patients with autoimmune hepatitis (Shan et al. 2023).

This may be due to the ability of zinc supplementation to inhibit of hepatic stellate cells activation, as reported in prior animal and in vitro studies (Ye et al. 2017; Xie et al. 2021).

TGF-β1 is a profibrotic cytokine known for stimulating the cellular synthesis and deposition of molecules within the extracellular matrix, including collagen types I, II, IV, and elastin. HSCs are the primary source of TGF-β1 although other cell types, such as macrophages, liver cells, and thrombocytes, also have the capacity to secrete this cytokine. The multifaceted involvement of various cell types underscores the intricate role of TGF-β1 in the fibrotic processes within the liver (Dewidar et al. 2019).

Additionally, zinc sulfate caused a non-significant decrease in serum TGF-β_1_ levels in the zinc group compared to the control which may be due to our patients being in early stages of liver fibrosis (F0, F1, F2) and the relatively small sample size.

The non-significant results of FIB-4 between the two groups indicate that the degree of liver fibrosis was minimal and varied between F0, F1, and F2, or may be caused by the limited number of patients.

Nevertheless, the notable decline in fibronectin and hyaluronic acid serum levels and significant negative correlations between zinc level and each of hyaluronic acid and TGF-B levels reflect the role of zinc supplementation to act against the mechanism of liver fibrosis and may represent what happened in the early stages of liver fibrosis in HCV.

Hence, our conclusions appear to be consistent with a few other reports which concluded that zinc concentrations reduced significantly with the progression of liver fibrosis and considered oral zinc supplementation to be beneficial in treating chronic hepatitis C (Hosui et al. 2018, 2021; Nishikawa et al. 2020; Kiouri et al. 2023).

One of the study's highlights is that it is the first clinical research focused on comparing the effectiveness of oral zinc sulfate tablets for liver fibrosis in Egyptian patients with hepatitis C virus who are receiving the DAAs combination of sofosbuvir 400 mg and daclatasvir 60 mg.

### Study restrictions

While our clinical research yielded beneficial results, it is important to acknowledge certain limitations. These include the study's open-label design, a single dose of 50 mg elemental zinc and a somewhat small sample size. Therefore, further larger-scale research with other doses of zinc and longer duration is necessary to confirm and expand on our findings.

## Conclusions

According to our research, administering zinc sulfate could potentially serve as a safe and efficient therapeutic strategy for managing hepatic fibrosis in individuals diagnosed with chronic hepatitis C virus, particularly those with mild and moderate hepatic fibrosis. However, given the study's limitations, ongoing research on a larger scale is essential to confirm and build upon these findings.

## Data Availability

The datasets analyzed during the current study are not publicly available due to [individual privacy could be compromised] but are available from the corresponding author on reasonable request.].
